# Impaired CpG Demethylation in Common Variable Immunodeficiency Associates With B Cell Phenotype and Proliferation Rate

**DOI:** 10.3389/fimmu.2019.00878

**Published:** 2019-04-24

**Authors:** Lucía del Pino-Molina, Javier Rodríguez-Ubreva, Juan Torres Canizales, María Coronel-Díaz, Marta Kulis, José I. Martín-Subero, Mirjam van der Burg, Esteban Ballestar, Eduardo López-Granados

**Affiliations:** ^1^Lymphocyte Pathophysiology in Immunodeficiencies Group, Department of Clinical Immunology, IdiPAZ Institute for Health Research, University Hospital La Paz, Madrid, Spain; ^2^Chromatin and Disease Group, Cancer Epigenetics and Biology Programme (PEBC), Bellvitge Biomedical Research Institute (IDIBELL), Barcelona, Spain; ^3^Fundació Clínic per a la Recerca Biomèdica, Barcelona, Spain; ^4^Departamento de Fundamentos Clínicos, Centro de Investigación Biomédica en Red de Cáncer, CIBERONC, Universitat de Barcelona, Barcelona, Spain; ^5^Institut d'Investigacions Biomèdiques August Pi i Sunyer (IDIBAPS), Barcelona, Spain; ^6^Institució Catalana de Recerca i Estudis Avançats (ICREA), Barcelona, Spain; ^7^Laboratory for Immunology, Department of Pediatrics, Leiden University Medical Center, Leiden, Netherlands

**Keywords:** common variable immunodeficiency, B cells, CpG, DNA methylation, B cell phenotype, proliferation rate

## Abstract

Common Variable Immunodeficiency (CVID) is characterized by impaired antibody production and poor terminal differentiation of the B cell compartment, yet its pathogenesis is still poorly understood. We first reported the occurrence of epigenetic alterations in CVID by high-throughput methylation analysis in CVID-discordant monozygotic twins. Data from a recent whole DNA methylome analysis throughout different stages of normal B cell differentiation allowed us to design a new experimental approach. We selected CpG sites for analysis based on two criteria: one, CpGs with potential association with the transcriptional status of relevant genes for B cell activation and differentiation; and two, CpGs that undergo significant demethylation from naïve to memory B cells in healthy individuals. DNA methylation was analyzed by bisulfite pyrosequencing of specific CpG sites in sorted naïve and memory B cell subsets from CVID patients and healthy donors. We observed impaired demethylation in two thirds of the selected CpGs in CVID memory B cells, in genes that govern B cell-specific processes or participate in B cell signaling. The degree of demethylation impairment associated with the extent of the memory B cell reduction. The impaired demethylation in such functionally relevant genes as *AICDA* in switched memory B cells correlated with a lower proliferative rate. Our new results reinforce the hypothesis of altered demethylation during B cell differentiation as a contributing pathogenic mechanism to the impairment of B cell function and maturation in CVID. In particular, deregulated epigenetic control of *AICDA* could play a role in the defective establishment of a post-germinal center B cell compartment in CVID.

## Key Points

- B cell key genes display defective demethylation in selected CpG sites in the transition from naïve to switched memory B cells in CVID, which might contribute to terminal B cell defects.
- Impaired demethylation is associated with the reduction of memory B cells in CVID patients.

## Introduction

Common Variable Immunodeficiency (CVID) comprises a group of primary syndromes of antibody deficiencies and constitutes the most frequent type of symptomatic primary immunodeficiency (PID), and yet it is not very well-understood. CVID patients are characterized by markedly decreased IgG with IgA, IgM, or both, defective specific antibody formation or decreased memory B cell counts. The majority of patients suffer recurrent bacterial infections, and many present severe complications such as autoimmune cytopenias, lymphoproliferative disorders, enteropathy, and malignancies, which suggests profound immune deregulation (1). All CVID patients present some degree of impaired differentiation of the B cell compartment, mostly normal percentage of circulating B cells, but markedly reduced memory B cells and absent plasma cells (2). Maturation and terminal specialization of B cells require the integration of molecular signals, driven by transcription factors and consolidated by epigenetic marks, which lead to initiation and maintenance of stage-dependent transcriptional programs (3). The epigenome refers to heritable modifications that influence gene expression but do not change the DNA sequence, such as DNA methylation and histone modifications (3, 4). DNA methylation consists of the addition of a methyl group to the 5′ position of a cytosine followed by a guanine, known as CpG dinucleotides. This modification is mediated by enzymes of the DNA methyltransferase (DNMT) family (5, 6). In some cases, CpG dinucleotides are clustered in regions termed CpG islands, many of which are located near gene promoters and their methylation generally associates with gene silencing. In contrast, isolated CpGs associate with different expression outcomes depending on their genomic location and chromatin context (3). The epigenome of B cells is dynamic, and it changes during differentiation. During the early steps of B cell differentiation in bone marrow, profound DNA demethylation is associated with the expression of B-specific lineage transcription factors such as E2A, EBF, and Pax5 (7–9). Upon antigen encounter, peripheral mature B cells undergo the germinal center (GC) reaction, in which somatic hypermutation (SHM) and class switch recombination (CSR) occurs. These processes are associated with a high replication rate and extensive demethylation (6, 10, 11). Finally DNA methylation changes are required for the establishment of long-lived memory and plasma B cells, which are essential for the maintenance of specific antigen responses (12–14).

CVID is mostly sporadic and the onset can occur at any age, with increased incidence at early adulthood. Progressive immunological deterioration can be traced occasionally before diagnosis is established. Isolated IgA deficiency can coexist in families with CVID members, and progression from one disease to another has been described. The general consensus is that CVID pathogenesis is complex and multifactorial, and only in 10–15% of the cases appears to be associated with a monogenic defect (15). Several genes harboring pathogenic or predisposing mutations have been associated with CVID, altering signaling pathways or molecular events relevant for B cell function and antibody formation. Initially, these genes include *CD19* (16)*, CD20* (17)*, CD21* (18)*, CD81* (19)*, ICOS* (20)*, TACI* (21)*, BAFFR* (22), however, recently more genes have been associated with CVID such as *TWEAK, CD27, IL21, IL21R, LRBA, CTLA4, PI3KCD, IKAROS, NFKB1, NFKB2, PRKCD, PIK3R1, VAV1, RAC2, BLK*, and *IRF2BP2* (23–25). Although new predisposing genes will surely be identified, it seems unlikely that a yet unknown single gene defect could account for the etiology of the genetically undiagnosed CVID patients. Therefore, although a predisposing genetic background seems plausible, immunological and clinical penetrance could depend on additional pathogenic mechanisms in most CVID patients (15).

The uncommon epidemiology and complex pathogenesis of CVID led us to explore new mechanisms that could impair relevant gene expression for terminal B cell function, other than in-born variations in DNA sequence. In a previous study (26), we reported, for the first time, the existence of aberrant DNA methylation in CVID B cells. Specifically, high-throughput DNA methylation analysis in B cells from a pair of CVID discordant monozygotic twins revealed a predominant impairment of DNA demethylation in critical genes for B cell biology. In addition, analysis of the DNA methylation profiles of sorted naïve, unswitched and switched memory B cells from a cohort of CVID patients revealed impaired DNA demethylation during naïve to memory B cell transition.

The most comprehensive study of DNA methylome variation during physiological human B cell maturation has recently been published by Kulis et al. (27), who, performing whole-genome bisulfite sequencing (WGBS) analysis, generated unbiased methylation maps of several sorted subpopulations spanning the entire B cell differentiation pathway in healthy individuals.

In this work, we expand our initial observation, and provide stronger evidence, by focusing our analysis on selected CpG sites near transcription start sites of genes that are relevant for late B cell differentiation. These CpG sites were selected from the study by Kulis et al. (27), and displayed significant demethylation in memory B cells compared to naïve B cells from healthy individuals. The list of genes include membrane receptors promoting survival, signaling mediators for cycle progression, activators of transcription factors, and genes involved in CSR and SHM. By using this approach, we confirmed the impaired demethylation in CVID memory B cells for most of the CpG sites analyzed. Our new results reinforce the hypothesis of a defective demethylation that associates with the functional and maturational impairment of memory B cells in CVID.

## Materials and Methods

### Patient Clinical and Immunological Study

Peripheral blood was obtained from 23 CVID patients (according to ESID criteria) and 17 healthy donors at La Paz University Hospital, after informed consent was obtained. The study was approved by the Ethics Committee of the Hospital, and adhered to the principles of the Declaration of Helsinki. Clinical data were obtained from hospital records.

B cell phenotype was performed staining whole blood with CD19 Percp Cy5.5, CD27 FITC, CD38 FITC (BD Biosciences) IgM Alexa Fluor 647 (Jackson Inmuno Research), and IgD PE (Southern Biotech) antibodies and analyzed on a FACS Canto flow cytometer (BD Biosciences).

Peripheral blood mononuclear cells (PBMCs) were obtained after Ficoll gradient centrifugation. Naïve (CD19^+^IgD^+^CD27^−^), unswitched memory USm (CD19^+^IgD^+^CD27^+^) and switched memory Sm (CD19^+^IgD^−^CD27^+^) B cells were sorted from viable PBMCs after staining with CD19 FITC (4G7BD), Ig D PE (Southern Biotech), CD27 BV421 (M-T271, BD) antibodies in a FACS Aria (BD Biosciences). Purity was confirmed to be >95% for selected fractions. Sorted cells were pelleted and stored at −80°C. Further details of the sorting strategy are depicted in Supplementary Figure 1.

### Candidate CpGs and Gene Selection for Methylation Analysis

We obtained the raw data available from the methylome analysis performed on several B cell subpopulations by Kulis et al. (27). We selected CpGs located at the promoter regions of several genes relevant for the B cell biology for analysis based on strict criteria: > 25% of difference in methylation in naïve (CD19^+^IgD^+^CD27^−^) vs. memory B cells (CD19^+^CD27^+^ IgA^+^ or IgG^+^). We considered a CpG site to be hypomethylated status when <0.5-fold was observed in the transition from naive to switch memory B cells. To increase the potential influence of selected CpGs on the transcriptional activity of the nearby genes, we included an extra requirement the proximity to at least two other CpGs with similar requisites for their methylation status in näive and memory B cells. We used the UCSC Genome Browser database (GRCh 37/hg 19) to precisely define the location of a given CpGs site to nearby genes (Supplementary Figure 2).

We selected nine CpG sites at the promoters of nine functionally relevant B cell genes (*AKT, TNFRSF13C, AICDA, BCL-6, BCL-10, FOXO1, MALT1, NFKB2*, and *STAT3*) (Supplementary Table 1 and Supplementary Figure 2). Each CpG was interrogated for its methylation status in sorted cell fractions of naïve, unswitched and switched memory B cells from CVID patients and healthy donors.

### Bisulfite-Modified DNA Pyrosequencing

We extracted DNA with *All prep DNA/RNA micro kit* (Qiagen) and bisulfite-converted using the *EZ-96 DNA methylation kit* (Zymo Research, Orange, CA, USA). We generated biotinylated amplicons for each CpG with specific primers (Sigma Aldrich) by PCR using the *Immolase DNA polymerase* (Bioline). Primers were designed with the PyroMark Assay Design Software (Qiagen version 2.0.01.15) (Supplementary Table 2). Pyrosequencing reactions were performed and DNA methylation quantified on a Pyromark Q96 MDsystem (Qiagen). Results from bisulfite pyrosequencing are presented as the percentage of methylation.

### KRECs and Igκ REHMA Assay

We inferred the replication history of naïve, unswitched and switched memory B cells from the same patients and healthy controls than in the DNA methylation study. To this end, we used the κ–deleting recombination excision circle (KREC) assay, as described by van Zelm et al. (28). The replication history is estimated by the ratio between genomic coding joints and signal joints on KRECs of the Igκ intron RSS-K deleting rearrangement, quantified by Real Time PCR using specific primers and probes (28), applying the ΔCT sample = Ct (signal joint)–Ct (coding joint) formula. The differences in Ct values is a measure for the number of cell divisions in B cells subsets that have undergone on average.

The extension of somatic hypermutation (SHM) in the memory B cell subset was measured using the Igκ-restriction enzyme hot spot mutation assay (IgκREHMA). This method described by van Zelm et al. (28) analyses the percentage of rearranged Vκ3-20 (VκA27) gene segments that contain somatic hypermutations in a specific sequence motif. Briefly, a PCR was performed on genomic DNA using an HEX-coupled Vκ3-20 intron forward primer and 2 FAM-coupled Jκreverse primers recognizing all 5 Jκ gene segments. PCR products of 500 bp were digested by restriction enzymes Fnu4HI and Kpn1, and the digested products run on an ABI 3130*xl* capillary sequencer (Applied Biosystems). Mutated gene products containing SHM generate 262 bp fragments, whereas unmutated 244 or 247 fragments. To assess the percentage of mutated alleles the calculation is:

$$\begin{array}{l} \%\text{ mutates alleles}=\frac{\text{peak height mutated fragment }\left(262\text{ pb}\right)}{\text{peak height unmutated fragments }\left(244\text{ and }247\text{ bp}\right)} \end{array}$$

### Statistical Analysis

The methylation status in B cell subsets from CVID patients and controls was analyzed and graphs were created with the GraphPadPrism 6.0 Software (GraphPad software, San Diego, California, USA). Two group comparisons were performed using *Mann Whitney test*, and Spearman's coefficient was used to determine correlation. Statistically significant was considered with *p* < 0.05 (^*^p < 0.05; ^**^p < 0.01; ^***^p < 0.001; ^****^p < 0.0001).

## Results

### Methylation Status of CpG Sites Near Promoters of Key B Cell Genes in CVID

In order to reassess and accumulate supportive data for the potential pathogenic relevance of impaired DNA demethylation in the generation or maintenance of the memory B cell compartment in most CVID patients (26), we applied an experimental strategy based on the analysis of selected CpGs sites with significantly differential DNA methylation in memory compared with naïve B cells in healthy individuals (27) (see Methods). The precise genomic location of those CpGs in relation to the nearest gene is depicted in Supplementary Figure 2 and their methylation status in the WGBS data from Kulis et al. is compiled in Supplementary Table 1.

All selected CpG sites had lower levels of DNA methylation in memory B cells compared to naïve B cells in healthy donors, and were located at critical genes involved in B cell differentiation and activation (*AKT1, FOXO1)* (29–33), NF-κB dependent regulation of B cell survival (*BCL-10, MALT1, NFKB2)* (34–36), JAK/STAT signaling (*STAT3)* (37, 38), B cell survival receptors *(BAFF-R)* (39, 40), CSR, SHM, and GC reaction (*AICDA, BCL-6*) (41–44) (Supplementary Figure 3).

We determined the DNA methylation levels of the aforementioned CpG sites in sorted fractions of naïve (CD19^+^IgD^+^CD27^−^), unswitched memory (CD19^+^IgD^+^CD27^+^) and switched memory (CD19^+^IgD^−^CD27^+^) B cells from a total of 23 CVID patients and 17 healthy donors by pyrosequencing of bisulfite-modified DNA.

**Figure 1:**
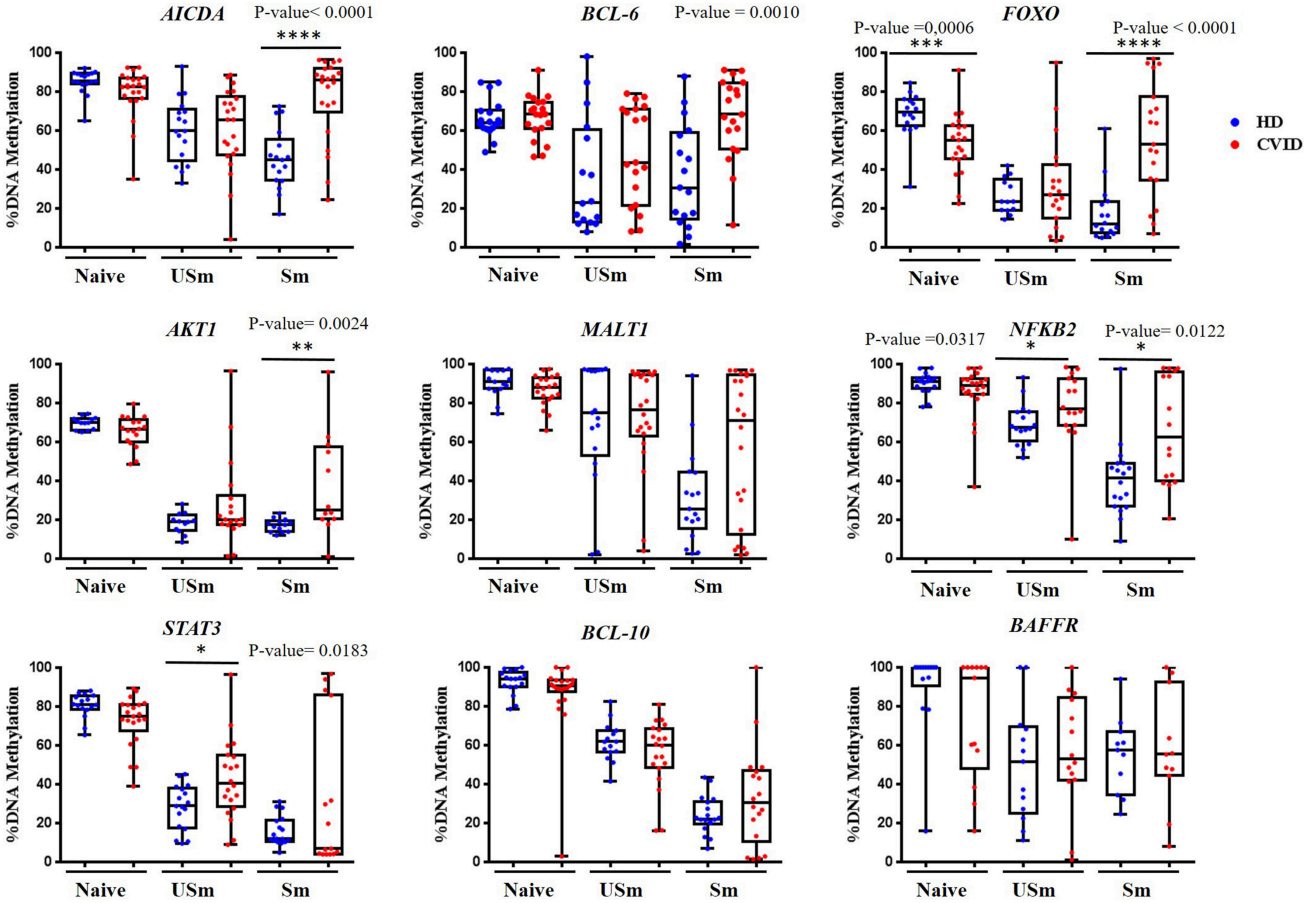
Percentage of DNA methylation obtained by pyrosequencing for the selected CpG in different genes, represented as box and whiskers, in the B cell subsets naïve, unswitched (USm) and switched (Sm) memory B cells from healthy donors (in blue) and CVID patients (in red). Box represent mean with minimum to maximum. *P*-value of statistically significant differences by Mann-Whitney test are included (*p < 0.05; **p < 0.01; ***p < 0.001; ****p < 0.0001).

Our analysis confirmed previous data by Kulis et al. (27) as all selected CpGs had higher levels of methylation in naïve B cells than class-switched memory B cells in healthy individuals (Figure 1). For unswitched memory B cells, the methylation levels for the selected CpGs were between naïve and switched memory B cells, supporting previous observations (45). Unswitched memory B cells expressing surface IgM can represent either alternative memory B cell differentiated from naïve B cells or initial stages in memory B cell differentiation. Furthermore, unswitched memory B cells can be generated both in a germinal center-independent and dependent reaction (46–48).

For all but one selected CpGs, CVID naïve B cells presented similar levels of methylation to healthy controls (Figure 1). Specifically, the CpG site at the *FOXO 1* gene displayed a significant decreased mean level of methylation in naïve B cells from CVID patients compared with controls (*p* = 0.0006; Figure 1).

The analysis of the methylation status of the selected CpG sites in memory B cells from CVID patients revealed three major different patterns. First, defective demethylation in switched memory B cells from CVID patients were observed compared with healthy counterparts (Figure 1). This pattern was observed in CpG sites at the *AICDA, BCL-6*, and *AKT1* genes, which, in switched memory B cells, displayed higher methylation in CVID patients compared with healthy donors (*p* < 0.0001, *p* = 0.0010, and *p* = 0.0024, respectively). Interestingly, although the CpG site at the *FOXO1* gene was hypomethylated in CVID naïve B cells, CVID switched memory B cells presented significantly higher methylation with respect to control (*p* < 0.0001). Second, defective demethylation was observed in both unswitched and switched memory B cells from CVID patients compared with healthy controls (Figure 1). This pattern was observed for the selected CpG at the *NFKB2* gene (*p* = 0.0317 and *p* = 0.0122, respectively). For the CpG site at the *STAT3* promoter, CVID unswitched memory B cells showed increased mean levels of methylation in comparison to healthy donors (*p* = 0.0183). Switched memory B cells presented dispersed methylation levels in the CpG at *STAT3* among CVID, with strikingly high levels of methylation in a subgroup of patients. Third, CpGs at the *MALT1, BCL-10*, and *BAFF-R* genes did not show significant differences neither in CVID unswitched memory nor in switched memory B cells (Figure 1) as compared to controls.

Therefore, impaired demethylation is predominantly observed in switched memory B cells from CVID, but is not a general phenomenon for all interrogated CpGs.

### Impairment of DNA Demethylation Associates With B Cell Phenotype in CVID Patients

**Figure 2:**
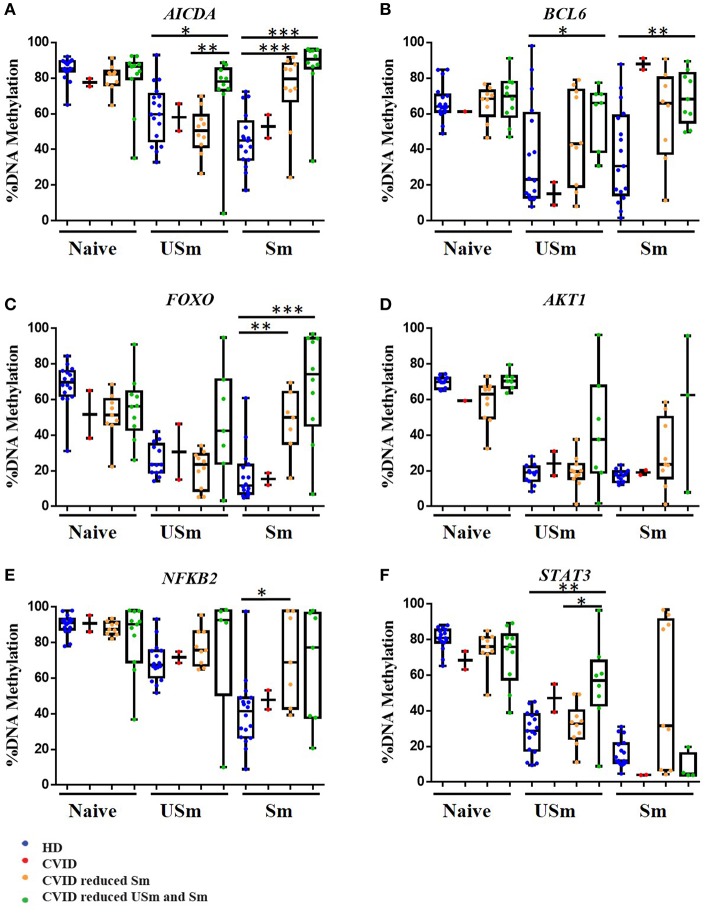
DNA methylation levels for the selected CpG in different genes, in the naïve, unswitched (USm) and switched (Sm) memory B cells represented as percentage in box and whiskers. HD are represented in blue. CVID patients with normal numbers of unswitched (USm) and switched (Sm) memory B cells in red, CVID patients with normal numbers of unswitched (USm) memory B cells but reduced switched (Sm) memory B cells in orange; in green CVID patients with reduced numbers of both unswitched (USm) and switched (Sm) memory B cells. Box represent mean with minimum to maximum. The *P*-value is shown for the cases with a statistically significant difference by Mann Whitney test (*p < 0.05; **p < 0.01; ***p < 0.001).

We next explored whether the impaired demethylation observed in selected CpGs present any correlation with the decreased numbers of unswitched and switched memory B cells in CVID patients. The reduction of memory B cells has been recently accepted as one of the diagnosis criteria for CVID. In the *Euroclass trial* (2) normal ranges for percentages of unswitched memory (7.2–30.8%) and switched memory (6.5–29.2%) B cells were established. Based on that strategy, we sub-classified our cohort of patients into three subgroups: CVID patients with normal numbers of unswitched (USm) and switched (Sm) memory B cells; CVID patients with normal numbers of unswitched (USm) memory B cells but reduced switched (Sm) memory B cells; and CVID patients with reduced numbers of both unswitched (USm) and switched (Sm) memory B cells. To analyze a possible relationship between the increased methylation of some CpGs and the number of memory B cells, we represented the results of those CpGs which showed significantly higher methylation levels in CVID memory B cells, but in this case subdividing patients according to the memory B cell phenotype (Figure 2).

Interestingly, CVID patients with normal percentages of unswitched memory and switched memory B cells had similar methylation levels than healthy counterparts for CpGs at the *AICDA, FOXO, AKT1, NFKB*2, and *STAT3* genes, in which these CpGs underwent demethylation to a similar extent to B cells from healthy individuals. The one exception was the CpG site at the *BCL-6* gene. This might suggest that impaired demethylation of *BCL6* (Figure 2B) in memory B cells could have an impact in the later step of differentiation toward plasma cells, a common feature to all CVID regardless the presence of switched memory B cells.

In addition, subclassification according to B cell phenotype revealed that patients with reduction of both unswitched and switched memory B cell compartments presented statistically significant hypermethylation of CpGs in *AICDA* and *BCL6* in unswitched memory B cells (*p* = 0.0110 and *p* = 0.0469, respectively; Figures 2A,B). These results suggest that defective acquisition of epigenetic and transcriptional status of certain B cell-specific genes, especially *AICDA* and *BCL6*, might be associated with the failure to produce or maintain normal levels of all type of memory and plasma B cells.

**Figure 3:**
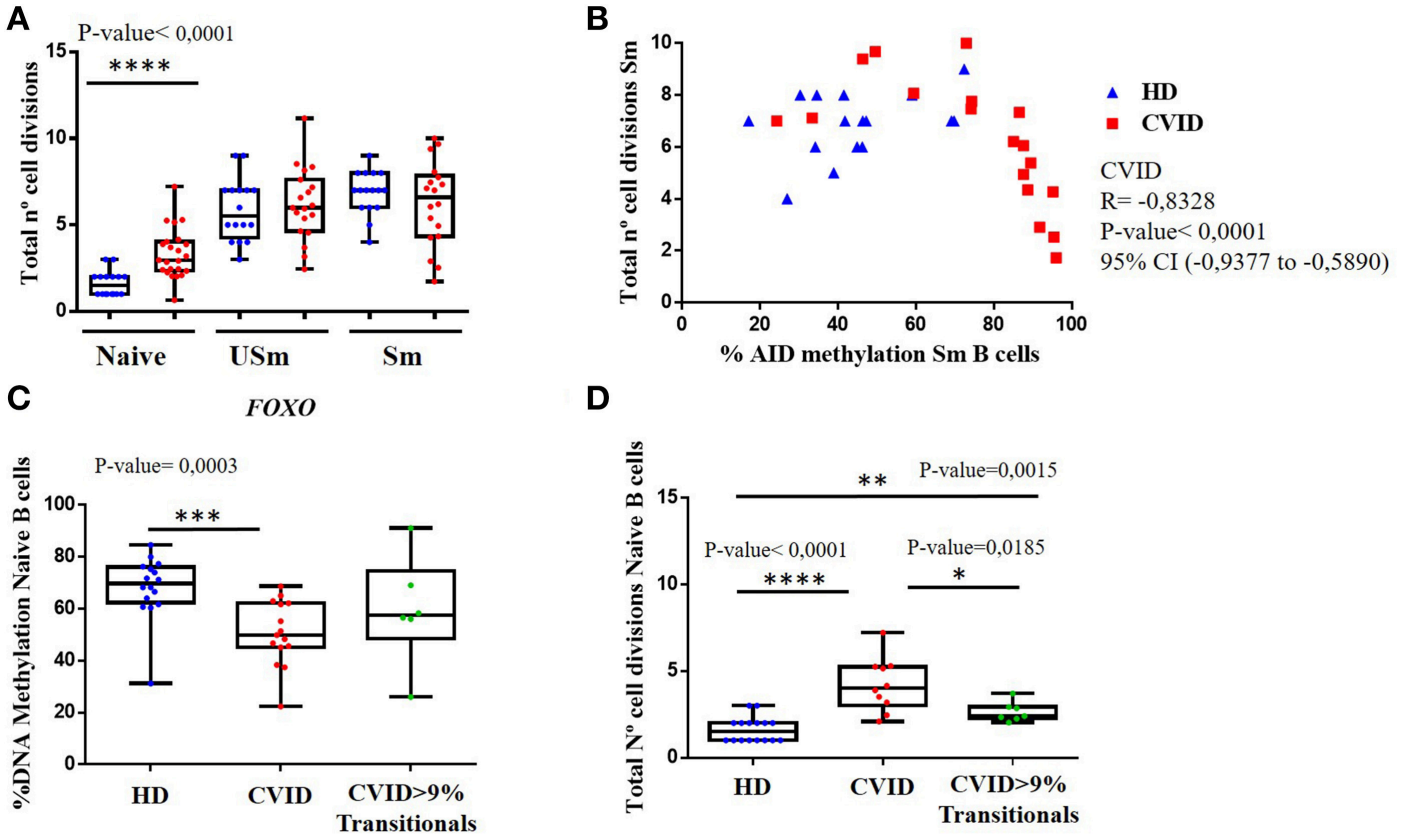
**(A)** Total number of cell divisions calculated by KREC assay, for the different B cell populations, naïve, unswitched (USm) and switched (Sm) memory B cells in HD (blue) and CVID patients (red). **(B)** Correlation between numbers of cell divisions in switched memory (Sm) B cells and the corresponding percentage of methylation of the selected CpG in *AICDA* in healthy donors (blue) and CVID patients (red). Spearman correlation test was used in CVID patients Spearman coeficient = −0.8328, *p* < 0.0001, 95% confidence interval (−0.9377 to −0.5890) in HD Spearman coeficient = 0.3130, *p* = 0.2367, 95% confidence interval (−0.2317 to 0.7082). **(C)** Box and whiskers showing the percentage of DNA methylation in naïve B cells obtained by pyrosequencing for the selected CpG in *FOXO* in HD (blue), CVID without expansion of transitional B cells (red) and patients with >9% transitional B cells (green). **(D)** Total number of cell divisions calculated by KREC assay in naïve B cells in HD (blue), CVID without expansion of transitional B cells (red) and patients with >9% transitional B cells (green). *P*-value of statistically significant differences by Mann-Whitney test are included (*p < 0.05; **p < 0.01; ***p < 0.001; ****p < 0.0001).

As indicated above, only the CpG at the *FOXO1* gene showed differential methylation levels in CVID naïve B cells when compared to healthy controls. In fact, according to the *Euroclass trial* (2), a subgroup of patients displayed increased percentages of transitional B cells that correlated with lymphoproliferative complications. Hence, we reanalysed the methylation levels for this CpG site in CVID patients based on the presence or absence of transitional B cells, and found that average methylation at this site in naïve B cells is significantly lower in patients without increased transitional B cells (Figure 3C).

### B Cell Proliferation Associates With Defects in Acquiring Correct DNA Methylation Changes

To identify possible factors along the B cell differentiation process that could contribute to the impaired demethylation observed in CVID memory B cells, we assessed the replication history, by KREC assay (28), in sorted B cell subpopulations from CVID patients and controls, utilizing the same DNA samples as the methylation analysis.

When we compared the total number of cell divisions in the different sorted B cells populations, we observed that naïve B cells from CVID patients had experienced a significantly higher number of divisions than those from healthy donors (*p* < 0.0001), (Figure 3A). In contrast, there were no significant differences in the accumulated number of B cell divisions in the unswitched and switched memory B cells between CVID patients and healthy controls. These results suggest that CVID B cells might have experienced an extra rate of proliferation at the naïve stage, perhaps rendering less cumulative opportunities for cell division through the GC reaction.

We next evaluated if the impaired CpG demethylation observed in CVID switched memory B cells could be related to the proliferation rate. We did not observe a clear correlation in healthy controls for the CpGs studied. However, a significant negative correlation between higher methylation levels in switched memory B cells and lower number of cell divisions in switched memory B cells is observed in CVID patients for CpGs at the *AICDA* (Spearman correlation = −0.8328, *p* < 0.0001) gene (Figure 3B). Furthermore, this negative correlation between the number of cell divisions and the methylation level in *AICDA* is also observed in unswitched memory B cells from CVID patients (Spearman correlation = −0.5273, *p* = 0.203).

In addition, we also tested whether cell proliferation could be playing a role in the decreased methylation levels of the CpG site at the *FOXO1* gene in naïve B cells from CVID patients when compared to healthy controls. CVID patients without increased transitional B cells, that also presented significantly lower methylation levels (Figure 3C), showed significantly higher number of cells divisions in naïve B cells when compared with normal controls and naïve B cells from CVID patients with increased transitional B cells (Figure 3D).

### Reduced Somatic Hypermutation (SHM) in CVID Memory B Cells With Hypermethylated *AICDA*

A major event in the late B cell differentiation is the diversification of the GC response with the generation of different immunoglobulin isotypes and subclasses from the IgM^+^IgD^+^ naïve B cells through the class switch recombination (CSR) and the maturation of antigen affinity by somatic hypermutation (SHM). Both processes depend on the crucial action of Activation-Induced Cytidin Deaminase encoded by the *AICDA* gene. AID is virtually not expressed in naïve B cells and becomes upregulated upon B cell activation during the GC reaction.

**Figure 4:**
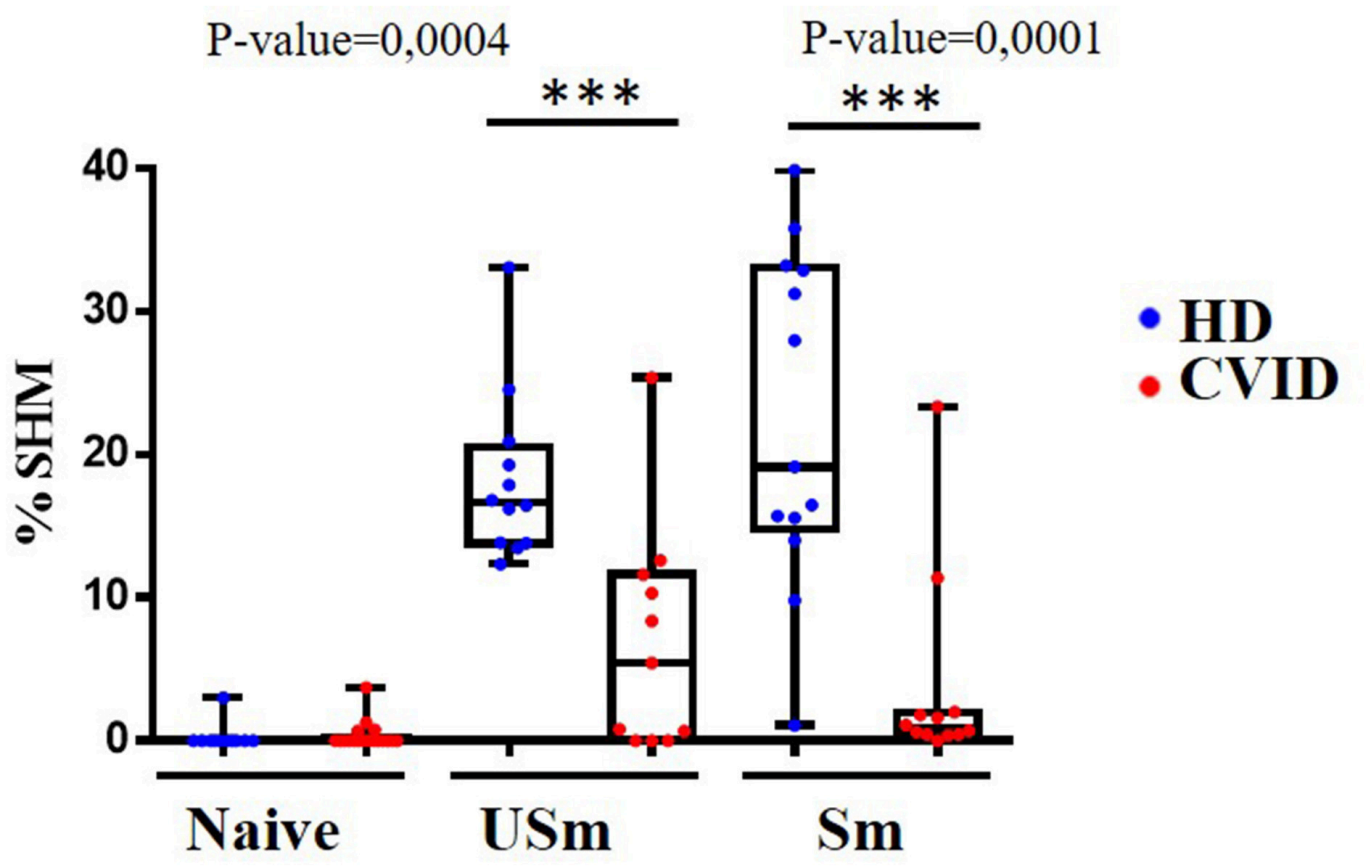
Frequency of SHM in naïve, unswitched (USm) and switched (Sm) memory B cells in healthy donors (blue) and CVID patients (red). By definition, naïve B cell do not undergo SHM, the frequency of SHM is severely reduced in USm and Sm in CVID patients. The *P*-value is shown for comparisons between HD and CVID patients with a statistically significant difference by Mann-Whitney test (***p < 0.001). Box and whiskers with minimum to maximum.

We perfomed IgκREHMA assay using the same DNA samples obtained from sorted populations of naïve, unswitched and switched memory B cells from CVID patients and controls. As expected, naïve B cells from both patients and controls showed virtual absence of SHM. Remarkably, SHM levels were significantly reduced in the unswitched and switched memory B cell subpopulations from CVID patients (*p* = 0.0004 and *p* = 0.0001, respectively) as shown in Figure 4. Reduced demethylation of *AICDA* in CVID individuals could be related to decreased AID expression in GC B cells of CVID patients, and subsequently contributing to decreased SHM in CVID memory B cells.

## Discussion

Aberrant DNA methylation has recently been shown to be a key feature in the deregulation of the B cell compartment of CVID patients (26). In this study, we dissected the divergent changes in DNA methylation of several relevant CpGs in the B cell subpopulations of CVID, providing further knowledge in understanding its etiology. We describe how several CpG sites, whose methylation levels decrease from naïve to memory B cells in healthy individuals (27), located at regulatory elements from key genes of B cell biology remain highly methylated in memory B cells from CVID patients. Most importantly, the severe reduction of memory B cells commonly seen in most CVID patients relates to the degree of impairment of demethylation in some genes, suggesting a role for their defective demethylation and the impaired generation of a competent memory B cell compartment. These new data, obtained by an alternative experimental approach to our original observation in a pair of CVID discordant monozygotic twins (26), reinforces the hypothesis that decreased demethylation is a possible contributing mechanism to the impaired B cell maturation in CVID patients.

Many genetically defined CVID patients present mutations in prototypical B cell genes, implying that intrinsic B cell defects could account for CVID. Paradoxically, pathogenic single gene variants seem to be only present in a minority of patients (15). B cell differentiation is promoted by activation of transcriptional programs governed by transcription factors and consolidated by epigenetic marks. One of the best characterized is DNA methylation, essential not only in B cells, but also in T cells, to modulate transcriptional programs in response to antigens as well as the acquisition of effector functions (49). DNA methylation contributes to transcriptional regulation through a number of mechanisms, including specific recruitment of methyl-CpG binding domain proteins, that form part of large chromatin remodeling complexes, and through the modulation of transcription factors binding (50).

Extensive DNA methylome remodeling along B cell development has been well-documented. At certain key stages, demethylation plays a fundamental role. Early B cell progenitors experience DNA demethylation concurrently with an increase in expression of essential transcription factors E2A, EBF, and Pax5 (3). During the GC reaction B cells have a high replication rate that couples with genome-wide demethylation. DNMTs and TET enzymes are responsible for the respective incorporation and removal of methyl groups to cytosines. On the other hand, passive loss of DNA methylation in heterochromatic late replication regions is achieved by DNA replication and high proliferative rates (3, 51). Division-dependent epigenomic remodeling and DNA demethylation has been studied in the terminal differentiation of B cells into plasma cells, highlighting the importance of demethylation, increased gene expression and the fact that B cells require a number of cell divisions to successfully reach the stage of plasma cells driven by a specific transcriptional program (13, 14). Nevertheless, the so-called epigenetic memory maintains transcription factor activity in more differentiated cell subsets, which, in memory and plasma B cells, drives their long lifespan and ability to generate specific antigenic responses (12, 27). Therefore, subtle alterations in epigenetic marks could have a critical impact in memory transcription profiles.

In CVID patients, six out of nine (66%) selected CpGs (*AICDA, BCL6, FOXO, AKT1, NFKB2*, and *STAT3*) showed significant hypermethylation in switched memory B cells in comparison to healthy donors. Impaired demethylation is therefore not detected for all CpGs studied, suggesting that this status is not related to an unspecific drift or generalized aberrancy of circulating cells.

*AICDA* is a key regulator of terminal B cell activation. In naïve B cells *AICDA* is epigenetically repressed, and the progression to the next maturation stages associates with promoter demethylation, together with permissive histone modifications that favor an open chromatin state (10). *AICDA* hypomethylation and expression has been shown to be essential for CSR and SHM (6, 11, 52). Our results suggest that deregulated epigenetic control of *AICDA* during the GC reaction may play a role in the defective establishment of a post-germinal center B cell compartment in CVID. First, we observed impaired *AICDA* demethylation in switched memory B cells from CVID patients, in which this impairment correlated with the reduced number of switched memory B cells in CVID patients. This correlation was also observed for reduced unswitched B cells, which is in agreement with a role of AID in unswitched memory B cells that experienced less extensive but evident SHM. Therefore, as expected, a significant impact in reduced SHM is observed in both switched and unswitched memory B cells from CVID patients.

Regarding the possible impaired mechanism by which *AICDA* is not experiencing demethylation in CVID memory B cells, we observed how the proliferative rate, measured by the KREC assay, is lower in those switched memory B cells with the highest methylation levels for *AICDA*. Defective passive demethylation during late B-cell differentiation could therefore be related to proliferative rates in CVID B cells. In this respect, the total number of cell divisions in CVID memory B cells is similar to healthy counterparts, suggesting that a gross proliferative defect is not implied in the reduced demethylation. Intriguingly, naïve B cells from CVID patients accumulated significant higher mean number of cell divisions than controls. This allows the speculation that memory B cells from CVID patients might have experienced reduced number of cell divisions in the transition from the naïve to the memory stage during GC reaction, although this cannot be formally proven at single cell level. A plausible explanation for defective demethylation in memory B cells from CVID patients could be that impaired upstream activation precludes division coupled epigenomic remodeling.

An alternative explanation is that active demethylation mechanisms might be defective in CVID patients. Although, it is still a vivid debate, a role for *AICDA* in active demethylation has been explored in the context of B cell activation (44). Domínguez et al. investigated the epigenetic function of *AICDA* in germinal center B cells in AID deficient (Aicda^−/−^) mice, implicating AID in the loss of DNA methylation in targeted specific loci in germinal center B cells. AID dependent demethylation is coupled to the rate of SHM. However, residual hypomethylation was still observed in Aicda^−/−^ mice, suggesting other mechanisms involved in DNA demethylation, such as TET enzymes or passive demethylation by high proliferation rates. Therefore, the impaired demethylation of *AICDA* observed in CVID patients could be contributing not only to the decreased SHM in memory B cells but to further demethylation processes.

Other relevant genes for B cell differentiation showed impaired DNA demethylation and might impact on B cell deregulation presented in CVID patients. *BCL6*, the master regulator for GC reaction to repress genes implicated in B cell activation, is a negative regulator of cell cycle and inhibits B cell differentiation to memory and plasma cells by Blimp-1 repression (42, 53). *BCL6* is regulated by post translational modifications (44) and it has been reported to interact with histone deacetylases HDAC1 (10) to potentially repress transcription. In all, this reflects the interplay between the key transcription factors and the epigenetic machinery during B cell differentiation (6, 53, 54). Interestingly, CVID patients with normal memory B cell phenotype that showed similar methylation patterns as controls for most CpGs continued to display impaired demethylation of the *BCL6* gene, indicating that altered methylation in some patients could contribute to impaired plasma cell differentiation.

Significant hypermethylation of CpGs near two other relevant genes promoters, for B cell signaling and maturation, *STAT3* and *NF*κ*B2*, was also identified. *STAT3* has a relevant role in IL10 and IL21 signal transduction for naive B cell differentiation to memory and plasma cells (37, 38). *NF*κ*B2* murine knockdown and mutant models show altered structures in lymph nodes and spleen with dysfunctional germinal centers, as well as impaired plasma cell formation (55, 56). Furthermore, germline mutations in *NF*κ*B2* have been associated with CVID pathogenesis (57).

All CpG methylation analyses so far showed similar patterns of methylation in CVID and control naïve B cells. We have found a first exception for a CpG related to *FOXO*, which was hypomethylated in CVID naïve B cells. *FOXO* transcription factors regulate genes with functions in apoptosis, cell cycle, longevity and DNA repair (30, 31). The axis PI3K-Akt-Foxo1 and the strict regulation of this pathway is relevant for B cell survival, proliferation, activation and terminal B cell differentiation (32, 33, 58).

Our new results of increased selective hypermethylation in functionally relevant genes for terminal B cell maturation reinforce the hypothesis of impaired demethylation as a mechanism affecting this process in CVID. Interestingly, gain of methylation was also originally reported in ICF syndrome, where hypermethylation was observed in genes associated with B cell differentiation despite a genome-wide loss of methylation attributed to a DNMT3 mutation. ICF syndrome patients can present a component of primary immunodeficiency immunologically similar to CVID (59).

In summary, our study suggests that impaired demethylation in the transition from naïve to memory B cells in CpGs at genes implicated in B cell signaling and survival (*STAT3, AKT1, FOXO*, and *NFKB2*), and at genes required for the GC reaction (*AICDA* and *BCL6)* might contribute to the terminal B cell defect in CVID patients. The intensity of impairment in demethylation is associated with the reduction of memory B cells in CVID patients. CVID is a complex disease not associated to single gene defects in many patients. Several accumulative B cells specific defects might be contributing to its pathogenesis. The potential implication of altered epigenetic control of terminal B lymphocyte differentiation is only at the dawn of its definition in CVID. Now we present novel insights that point out the possible implication of both passive, due to reduced proliferation rate between naive and switched memory B cells, and active demethylation mechanisms involving *AICDA* in the pathogenesis of CVID. Based on our data, we cannot conclude if defective demethylation is a primary pathogenic mechanism disturbing memory B cell differentiation, or a downstream contributing consequence of impaired activation or signaling in CVID memory B cells.

## Ethics Statement

This study was carried out in accordance with the recommendations of La Paz University Hospital committee with written informed consent from all subjects. All subjects gave written informed consent in accordance with the Declaration of Helsinki. The protocol was approved by La Paz University Hospital Committee.

## Author Contributions

LdP-M, JR-U, MvdB, EB, and EL-G designed research. LdP-M and JR-U performed experiments and analyzed data. JT and MC-D contributed to perform some experiments. MK and JM-S contributed with original data from whole genome bisulfite sequencing analysis. LdP-M, JR-U, EB, and EL-G wrote the paper.

### Conflict of Interest Statement

The authors declare that the research was conducted in the absence of any commercial or financial relationships that could be construed as a potential conflict of interest.

## Supplementary Material

Supplementary Figure 1 Sorting strategy. B cell subpopulations were sorted from PBMCs, lymphocytes were identified by forward (FSC) and scatter (SSC) properties. Total B cells were identified by CD19^+^, and classified according to IgD and CD27 expression into Naïve (CD19^+^IgD^+^CD27^−^), unswitched memory USm (CD19^+^IgD^+^CD27^+^) and switched memory Sm (CD19^+^IgD^−^CD27^+^) B cells.

Supplementary Figure 2 Representation of available methylation data of CpGs (in black) with the selected CpG in red. The Transcription Start Site (TSS) is indicated for each gene with the exons represented in blue boxes; and the CpG islands in green boxes. Log2 Ratio calculated as the % DNA methylation (memory B cells/naïve B cells).

Supplementary Figure 3 Selected genes in blue implication in B cell survival (*BAFFR*), PI3K activation pathway (*AKT1* and *FOXO*), NFκB canonical pathway (*BCL-10, MALT1*), NFκB alternative activation pathway (*NF*κ*B2*), JAK/ STAT signaling from cytokine receptors (*STAT3*), masterclass regulator of germinal center reaction (*BCL-6*) and *AID* for CSR and SHM in the germinal center.

Supplementary Table 1 Selected CpG in different genes, it is indicated the chromosome localization (Chr) and map info. From Kulis et al. we obtained the methylation status the mean of the two replicates in naive (N) and Memory (M) B cells. We performed the Difference (Mean N- Mean M) and the Ratio (Mean M/Mean N).

Supplementary Table 2 Primers for PCR amplification and pyrosequencing.
