# Supplementary material for: Impaired CpG Demethylation in Common Variable Immunodeficiency Associates With B Cell Phenotype and Proliferation Rate
Source: Front Immunol. 2019 Apr 24;10:878. doi: 10.3389/fimmu.2019.00878 (PMC6492528; doi:10.3389/fimmu.2019.00878)
Supplement: Supplementary Figure 1 — Sorting strategy. B cell subpopulations were sorted from PBMCs, lymphocytes were identified by forward (FSC) and scatter (SSC) properties. Total B cells were identified by CD19+, and classified according to IgD and CD27 expression into Naïve (CD19+IgD+CD27−), unswitched memory USm (CD19+IgD+CD27+) and switched memory Sm (CD19+IgD−CD27+) B cells. [file Data_Sheet_1.PDF]

Supplementary Figure 1

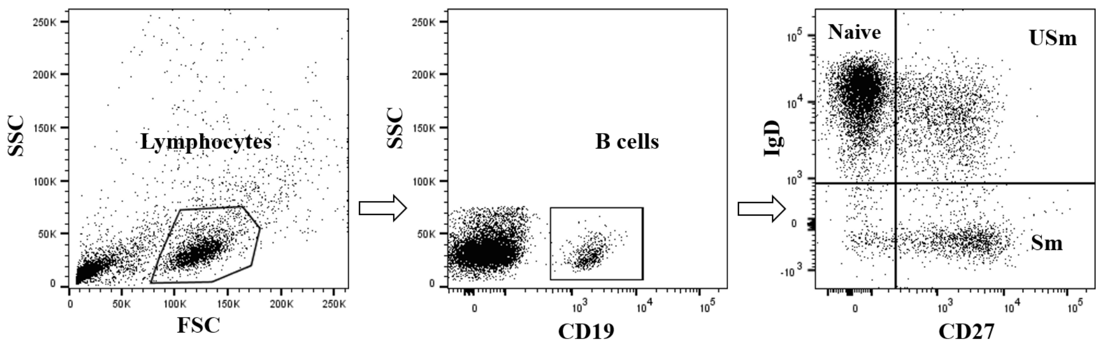

Supplementary Figure 2

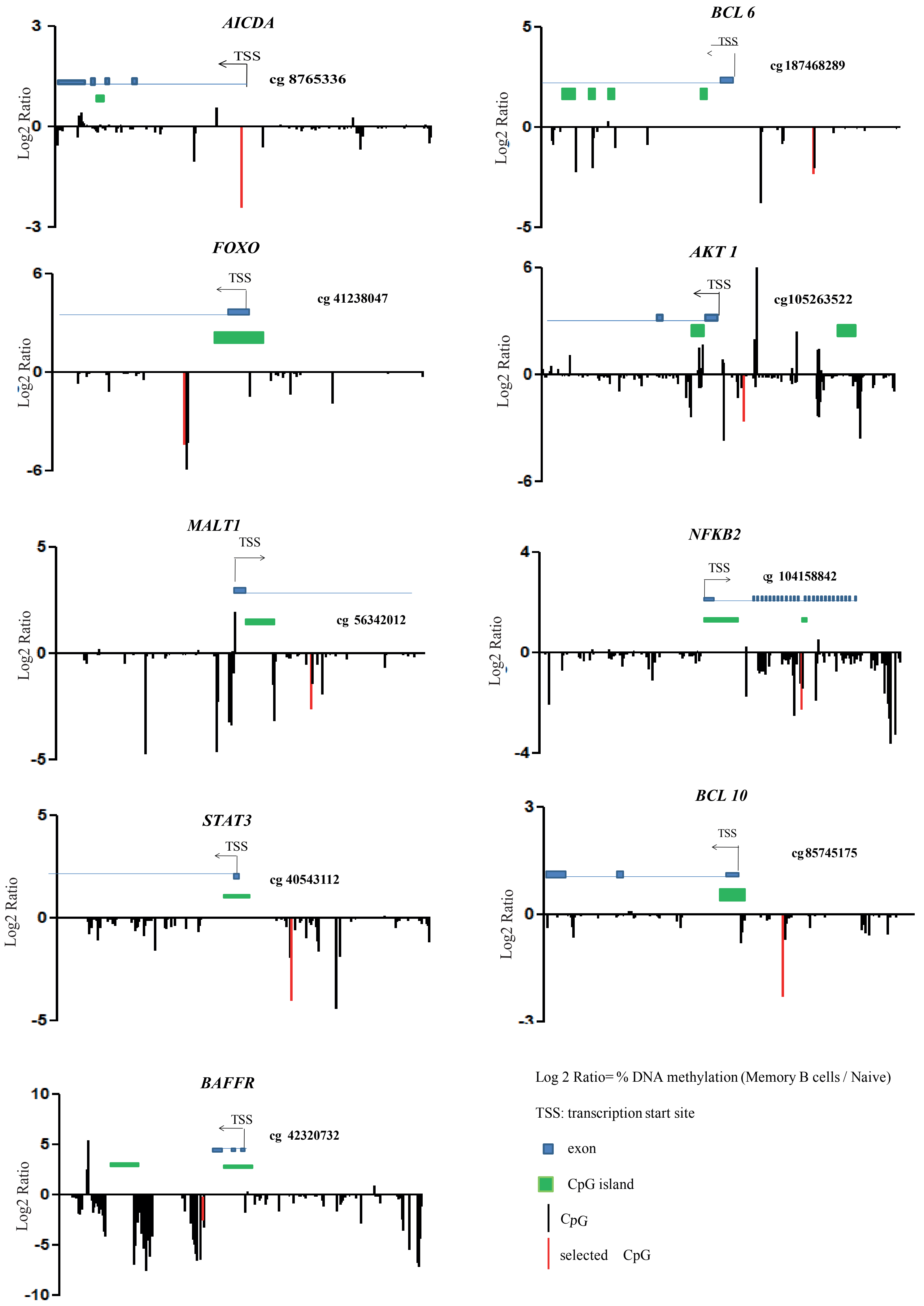

Supplementary Figure 3

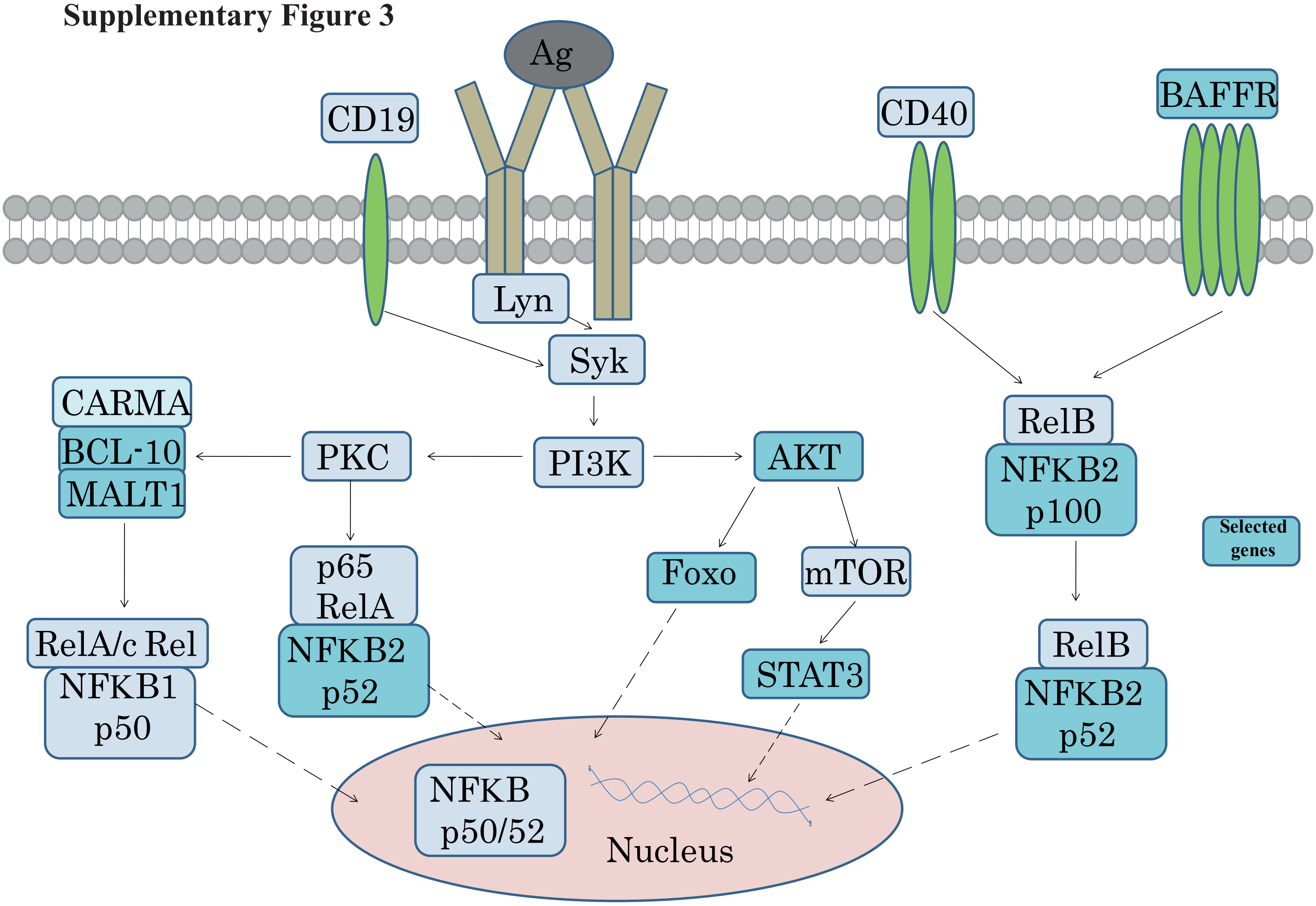

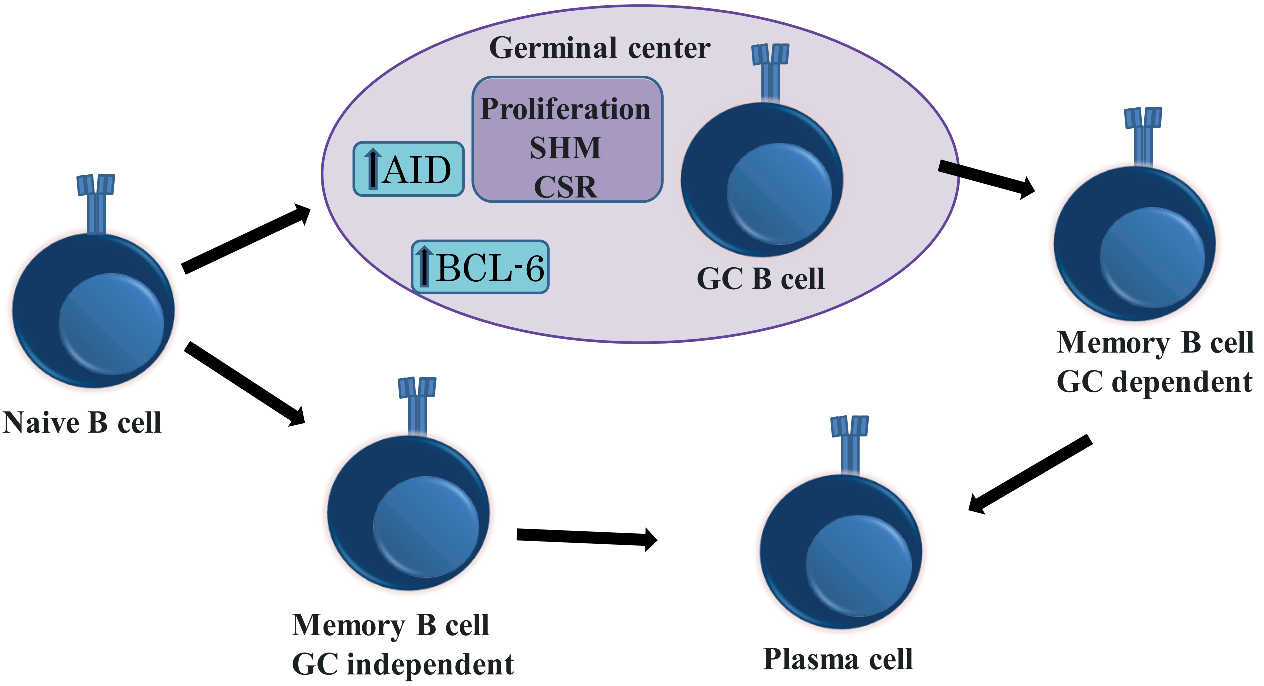

Supplementary table 1

	Chr	MAPINFO	Mean N	Mean M	Difference (Mean N- Mean M)	Ratio (Mean M/ Mean N)
AICDA	chr12	8765336	0.8770545	0.16275055	0.71430395	0.18556492
AKT1	chr14	105263522	0.72462	0.11752735	0.60709265	0.1621917
BCL10	chr1	85745175	0.875305	0.1778385	0.6974665	0.20317318
FOXO1	chr13	41238047	0.6171275	0.02840715	0.58872035	0.04603125
MALT1	chr18	56342012	0.848091	0.1348035	0.7132875	0.15894933
NFKB2	chr10	104158842	0.829262	0.173367	0.655895	0.20906179
STAT3	chr17	40543112	0.8718725	0.0526339	0.8192386	0.0603688
TNFRSF13C (BAFFR)	chr22	42320732	0.894724	0.1377905	0.7569335	0.15400336
BCL6	chr3	187468289	0.604177	0.1189405	0.4852365	0.19686367

Supplementary Table 2：Primers for PCR amplification and pyrosequencing

AICDA (map info 8765336)	Forward GTTTTGAGAGGAAGGTTAGTGTAA<br>Reverse (biotinylated) CAAAAAAACACTCTAAACACCACTAT<br>Sequencing AGGAAGGTTAGTGTAAT
AKT (map info 105263522)	Forward GAGTTATTTGGGGTTTTTTTTGGGATTG<br>Reverse (biotinylated) AATTCCTTCCTCCATCCTTACATA<br>Sequencing ATTGTTTTATTTGTTTATTGTTTTT
BCL6 (map info 187468289)	Forward AGTTATTATATTGTTATTTGAGGTTAGAAG<br>Reverse (biotinylated) TTTTTTTTTTCTCCCTACACTATACC<br>Sequencing GTTAGAAGATAGATATTTTGGT
BCL10 (85745175 map info)	Forward GGGTGGATGAGTAGAAAGAATA<br>Reverse (biotinylated) ATATAAAAAAACCRCCAAACTAAACACC<br>Sequencing AGAAAGAATATTTAGGGGA
FOXO1 (map info 41238047)	Forward GTATTAATTATAGGGATGATATTGTGTGT<br>Reverse (biotinylated) ACTAAACACCTTTTTAAAACATACCT<br>Sequencing AAAATAATTTTGGTGTTAATAATTG
MALT1 (map info 56342012)	Forward TTGGTGTTAAAGATAGGAATTATAGTGTG<br>Reverse (biotinylated) ACAAATCCAATAACTAAAAAAAACACTTC<br>Sequencing GTTTAGTTCRTGTGGGTGA
NFKB2 (map info 104158842)	Forward AGGAGTTTAAGATTAGTTTGGTTAATAG<br>Reverse (biotinylated) CTCAACTTACTACAACCTCTTATC<br>Sequencing AGTTGGGTATGGTGG
STAT3 (map info 40543112)	Forward AGGTTGAGGGAAGAGAATTTT<br>Reverse (biotinylated) CCTACCTTAACCTCCTAAATAATTAAAACT<br>Sequencing AGTAAAAATTAGTTAGTTATGGTG
BAFFR (map info 42320732)	Forward GTTTAGGTTGTAATTAGTTGGGGATTT<br>Reverse (biotinylated) AAATTTCACCATATTAACCAAAATAATCTC<br>Sequencing ATTTTAGTATTTTGGGAGG
